# Discrimination of double primary lung cancer from intrapulmonary metastasis by p53 gene mutation

**DOI:** 10.1038/sj.bjc.6690247

**Published:** 1999-03

**Authors:** D Matsuzoe, T Hideshima, K Ohshima, K Kawahara, T Shirakusa, A Kimura

**Affiliations:** Second Department of Surgery, School of Medicine, Fukuoka University, Jonanku, Fukuoka 814-0180, Japan; First Department of Pathology, School of Medicine, Fukuoka University, Fukuoka, Jonanku, 814-0180, Japan; Division of Adult Diseases, Medical Research Institute, Tokyo Medical and Dental University, Tokyo, Chiyodaku, 101-0062, Japan

**Keywords:** double lung cancer, p53, clonal marker, intrapulmonary metastasis

## Abstract

When multiple synchronous lung tumours are identified, discrimination of multicentric lung cancers from intrapulmonary metastases by clinical findings is often difficult. We used genetic alterations in p53 gene as a discrimination marker of double primary lung cancers from single lung cancer with intrapulmonary metastasis. Twenty of 861 patients with primary lung cancer who underwent lung resection were selected as subjects because they showed synchronous double solid tumours of the same histological type in the unilateral lung without distant metastases. In addition, they had been diagnosed as lung carcinoma with intrapulmonary metastasis by clinical and histological findings. DNAs were extracted from paraffin-embedded tissue of paired tumours from these 20 patients. Exons 5–9 of the p53 gene were examined for genetic alterations in the tumours by polymerase chain reaction, single-strand conformation polymorphism analysis and subsequent DNA sequencing analysis. Three different patterns in the distribution of p53 mutations in double lung tumours were observed: [A] mutation in only one of the tumours (four cases), [B] different mutations in the tumours (two cases), and [C] same mutation in both tumours (one case). The cases of [A] or [B] patterns could be classified as double primary lung cancers, while the case of the [C] pattern was suggested to be lung cancer with intrapulmonary metastasis. These results suggested that the multicentric cancers were more frequent than the intrapulmonary metastatic cancers in double cancer cases. © 1999 Cancer Research Campaign


British Journal of Cancer (1999) **79**(9/10), 1549–1552
© 1999 Cancer Research Campaign
Article no. bjoc.1998.0247

### Discrimination of double primary lung cancer from intrapulmonary metastasis by p53 gene mutation

**D Matsuzoe[1], T Hideshima[1], K Ohshima[2], K Kawahara[1], T Shirakusa[1] and A Kimura[3]**

[1]Second Department of Surgery and [2]First Department of Pathology, School of Medicine, Fukuoka University, 7-45-1 Nanakuma, Jonanku, Fukuoka 814-0180, Japan; [3]Division of Adult Diseases, Medical Research Institute, Tokyo Medical and Dental University, 2-3-10 Kanda surugadai, Chiyodaku, Tokyo 101-0062, Japan

**Summary** When multiple synchronous lung tumours are identified, discrimination of multicentric lung cancers from intrapulmonary metastases by clinical findings is often difficult. We used genetic alterations in p53 gene as a discrimination marker of double primary lung cancers from single lung cancer with intrapulmonary metastasis. Twenty of 861 patients with primary lung cancer who underwent lung resection were selected as subjects because they showed synchronous double solid tumours of the same histological type in the unilateral lung without distant metastases. In addition, they had been diagnosed as lung carcinoma with intrapulmonary metastasis by clinical and histological findings. DNAs were extracted from paraffin-embedded tissue of paired tumours from these 20 patients. Exons 5–9 of the p53 gene were examined for genetic alterations in the tumours by polymerase chain reaction, single-strand conformation polymorphism analysis and subsequent DNA sequencing analysis. Three different patterns in the distribution of p53 mutations in double lung tumours were observed: [A] mutation in only one of the tumours (four cases), [B] different mutations in the tumours (two cases), and [C] same mutation in both tumours (one case). The cases of [A] or [B] patterns could be classified as double primary lung cancers, while the case of the [C] pattern was suggested to be lung cancer with intrapulmonary metastasis. These results suggested that the multicentric cancers were more frequent than the intrapulmonary metastatic cancers in double cancer cases.

**Keywords:** double lung cancer; p53; clonal marker; intrapulmonary metastasis

The occurrence of multiple primary cancer within the same organ has been widely reported. The incidence of multiple lung cancers in reported clinical series ranges from 1% to 7% (Ferguson et al, 1985), and autopsy studies have revealed more accurately that the incidence of multiple primary tumours in the lung ranges from 3.5% to 14% (Mathisen et al, 1984). When we identify multiple synchronous lung tumours, differentiating multicentric lung cancers, a single lung cancer with intrapulmonary metastases, or pulmonary metastases from primary cancer in different organs can often be difficult, especially when multiple tumours are found in the same lobe. There are no specific clinical or radiologic features that can differentiate the multiple primary lung cancers and intrapulmonary metastases. Non-small cell lung cancer cases with intrapulmonary metastasis were divisible into two subgroups of patients, one with a good post-surgical prognosis and one with a poor outcome, and there is a possibility that pulmonary metastases with good prognosis may be multicentric lung cancers (Naruke et al, 1988; Nakajima et al, 1996). Several reports have suggested that the incidence of synchronous double primary lung cancer might be higher than predicted and they have advocated an aggressive treatment plan for double primary lung cancer cases (Ferguson et al, 1985; Mathisen et al, 1984; Kunitoh et al, 1992; Martini et al, 1975).

p53 mutation is one common genetic change in non-small cell lung cancers (Chiba et al, 1990; Caamano et al, 1991; Mitsudomi et al, 1992; Kishimoto et al, 1992). It has been demonstrated that the p53 mutation usually precedes metastasis and is conserved in metastases (Chung et al, 1993; Li et al, 1994; Zheng et al, 1994). On the other hand, the p53 mutation pattern has been used to distinguish primary tumour from metastasis and/or second primary tumour from recurrence in various organ tumours, such as hepatocellular carcinomas (Oda et al, 1992), ovarian cancers (Mock et al, 1992), urothelial carcinomas (Habuchi et al, 1993), and head and neck cancers (Gasparotto et al, 1995; Nees et al, 1993). With respect to the lung cancer, comparison of p53 mutations between primary lung cancer and metastatic lung tumour (Kandioler et al, 1996), primary lung tumour and metastases to other organs (Schlegel et al, 1992; Reichel et al, 1994), or second primary lung cancer and metastasis (Sozzi et al, 1995; Mitsudomi et al, 1997) has been reported. However, discrimination of multicentric lung cancers from pulmonary metastasis has never been examined in synchronous double lung tumour cases.

It is possible that synchronous double primary lung cancer has been misdiagnosed as single lung cancer with pulmonary metastasis in some cases. In this study, we examined 20 patients with synchronous double lung tumours diagnosed as primary lung cancer with intrapulmonary metastasis by clinical and histological findings to clarify the clonal origin of these tumours using p53 gene mutation as a clonal marker.

*Received 1 April 1998*
*Revised 11 September 1998*
*Accepted 29 September 1998*

*Correspondence to:* D Matsuzoe

### MATERIALS AND METHODS

### Patients and DNA extraction

Histological reports of patients who underwent lung resection for primary lung cancer at the Second Department of Surgery of

**Table 1** Oligonucleotide sequences used for p53 gene amplification and length of amplified fragments

| Exon | Amplification | Length (bp) |
|---|---|---|
| 5-f | s 5′-TGTCTCCTTCCTCTTCCT-3′ | 137 |
| | a 5′-TGTAGATGGCCATGGCG-3′ | |
| 5-l | s 5′-CTGTGGGTTGATTCCACA-3′ | 144 |
| | a 5′-CAGCCCCAGCTGCTCA-3′ | |
| 6 | s 5′-TGATTCCTCACTGATTGCTC-3′ | 160 |
| | a 5′-GAGACCCCAGTTGCAAACC-3′ | |
| 7 | s 5′-TCTTGGGCCTGTGTTATCTC-3′ | 171 |
| | a 5′-AGCAGGCCAGTGTGCAGG-3′ | |
| 8-f | s 5′-CTTACTGCCTCTTGCTTCTC-3′ | 122 |
| | a 5′-AGATTCTCTTCCTCTGTGCG-3′ | |
| 8-l | s 5′-TTTGAGGTGCGTGTTTGTGC-3′ | 135 |
| | a 5′-CGCTTCTTGTCCTGCTTGC-3′ | |
| 9 | s 5′-CTTTTATCACCTTTCCTTGCC-3′ | 137 |
| | a 5′-TCCACTTGATAAGAGGTCCC-3′ | |

f, first half; l, latter half; s, sense primer; a, antisense primer.

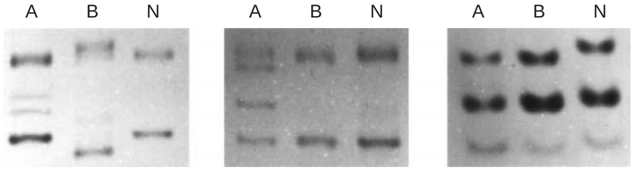

**Figure 1** Cases representative of three different distribution patterns of p53 mutations in double lung tumours. DNAs from different tumours of the same patient (A and B) and a normal control (N) were analysed. Case 9 showed different mutations in the tumours. Case 15 showed the mutation in only one tumour. Case 16 showed the same mutation in both tumours

Fukuoka University Hospital between the 1986 and 1996 were reviewed. Twenty of 861 patients were selected in accordance with the following criteria: they had synchronous double solid tumours (1) in the unilateral lung, (2) without distant metastases, (3) of the same histological type, and (4) diagnosed as intrapulmonary metastasis by clinical and histological findings. Paired tumours from these 20 patients were analysed. Sections of tumours 30-μm thick were prepared from each formalin-fixed, paraffin-embedded block after as much of the normal tissues around the tumour as possible were cut away. The sections were deparaffinized with three successive xylene extractions, rinsed once each with 100%, 90% and 70% ethanol, and air dried. Samples were incubated in 500 mM Tris-HCl (pH 8.0), 20 mM ethylenediamine tetra-acetic acid (EDTA), containing 1% sodium dodecyl sulphate and 1 mg $ml^{-1}$ proteinase K for 96 h at 42°C. DNAs were purified by extracting twice with one volume of phenol–chloroform–isoamylalcohol (25:24:1) and twice with one volume of chloroform–isoamylalcohol (24:1), precipitating with ethanol, rinsing twice with 70% ethanol and dissolved in 10 mM Tris-HCl, 1 mM EDTA (pH 8.0).

### Polymerase chain reaction, single-strand conformation polymorphism analysis and sequence analysis

Because most (95–98%) of the mutations in the p53 gene in human cancers have been found in exons 5–9, these exons were separately amplified by polymerase chain reaction (PCR) and subjected to single-strand conformation polymorphism (SSCP) analysis. The sequences of primers used to amplify exons 5–9 are given in Table 1. Because of the limited length of DNA extracted from formalin-fixed speciments, PCR products were sized less than 200 base pairs. Exons 5 and 8 were each divided into two overlapping fragments. Samples were electrophoresed in 10 or 12% non-denaturing polyacrylamide gels with and without 10% glycerol at room temperature. The DNA fragments were detected by silver staining with the Silver Stain ‘DAIICHI’ (Daiichi Pure Chemicals, Tokyo, Japan). PCR products showing abnormal SSCP band patterns were purified from a 2% agarose gel and cloned into

**Table 2** Mutations of the p53 gene in double lung tumours

| Case | Age (yr) | Sex | Tumour | Site | Size (cm) | Histology | p53 mutations: Tumour | | p53 mutations: Metastatic lymph node | |
|---|---|---|---|---|---|---|---|---|---|---|
| 4 | 77 | M | 4A | LU | 7.0 | Sq | Codon 158 | CGC → TGC | No metastasis | |
| | | | 4B | LU | 1.5 | Sq | Codon 138 | GCC → GTC | | |
| 9 | 68 | M | 9A | RU | 6.0 | Ad | Codon 233 | CAC → TAC | No metastasis | |
| | | | 9B | RU | 1.5 | Ad | Codon 260 | TCC → TCT | | |
| 10 | 74 | M | 10A | LU | 5.0 | Sq | Codon 237 | ATG → ATT | No metastasis | |
| | | | 10B | LU | 1.5 | Sq | No mutation | | | |
| 15 | 67 | M | 15A | RM | 4.5 | Ad | Codon 250 | CCC → CTC | No metastasis | |
| | | | 15B | RM | 2.0 | Ad | No mutation | | | |
| 16 | 64 | M | 16A | RU | 4.0 | Ad | Codon 268 | AAC → AAT | Codon 268 | AAC → AAT |
| | | | 16B | RU | 2.0 | Ad | Codon 268 | AAC → AAT | | |
| 19 | 62 | M | 19A | LU | 6.0 | Sq | No mutation | | Codon 250 | CCC → CTC |
| | | | 19B | LU | 1.5 | Sq | Codon 250 | CCC → CTC | | |
| 20 | 49 | F | 20A | LL | 4.0 | Sq | No mutation | | Codon 143 | GTG → GCG |
| | | | 20B | LU | 1.5 | Sq | Codon 143 | GTG → GCG | | |

M, male; F, female; LU, left upper lobe; RU, right upper lobe; RM, right middle lobe; LL, left lower lobe; Sq, squamous cell carcinoma; Ad, adenocarcinoma.

 *© Cancer Research Campaign 1999*

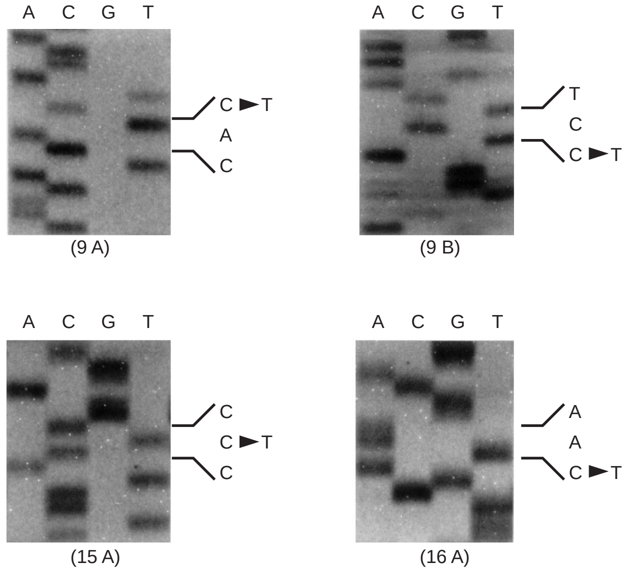

**Figure 2** Sequencing analysis of the p53 gene mutations in tumours: (9A) mutation in codon 233; (9B) mutation in codon 260; (15A) mutation in codon 250; (16A) mutation in codon 268

a TA cloning vector (Invitrogen, San Diego, USA). Each ligation reaction was transformed into competent JM 109 cells (Toyobo Osaka, Japan). The resulting colonies were picked up and then the colony containing mutated p53 was identified by PCR-SSCP analysis as described above. The plasmid DNA was isolated from each identified clone and sequenced by using the T7 Sequenase v2.0 DNA Sequencing Kit (Amersham, Ohio, USA).

### RESULTS

Seven out of 20 (35%) cases showed abnormally shifted bands of the p53 gene in at least one tumour in the SSCP analysis (Figure 1). No abnormality was found in the remaining 13 cases. Of the seven cases, both tumours were located in the same lobe in six cases and in different lobes in one case (Table 2). Histologically, four cases were squamous cell carcinomas and three cases were adenocarcinomas. Three cases (cases 4, 19 and 20) underwent pneumonectomy, and the others underwent lobectomy. Three patients died within 6 months after the operation (cases 4, 15 and 20). Only two patients survived more than 2 years (cases 10 and 19 for 6 years and 25 months respectively) after the operation.

Mutations in the p53 gene were confirmed by the DNA sequencing analysis of the PCR product (Figure 2). Four cases (cases 10, 15, 19 and 20) had p53 mutations in only one tumour. Two cases (cases 4 and 9) had different mutations specific to each tumour. One case (case 16) had the same mutation in both tumours. Three of seven cases had lymph node metastasis, and these nodules were analysed further. All of the metastatic lymph nodes were found to have the p53 gene mutations. In cases 19 and 20, the mutation in metastatic lymph node was identical to that in the tumour with p53 mutation. In case 16, both the pair of tumours and lymph node metastasis had the same mutation. A possibility of germline mutation in this case was excluded by certifying that no genetic changes were found in the normal lung tissue. In this study, we did not evaluate the p53 protein expression using immunohistochemistry.

### DISCUSSION

Clinically, human tumours can be divided into three groups: premalignant lesions, primary tumours and metastases. Normal cells or premalignant cells become malignant after accumulation of genetic alterations. The malignant cells have a growth advantage and begin clonal expansion to produce primary tumours. During expansion of the converted cells at the primary site, new clones with more malignant phenotypes appear along with additional genetic alterations in some of the converted cells (Yokota and Sugimura, 1993). Metastatic nodules are produced by highly selected and progressed cells that are more invasive and highly metastatic. They contain the genetic alterations including mutations in the p53 gene that are necessary to maintain the malignant phenotypes acquired during the progression. p53 mutation usually precedes metastasis and is conserved in metastases (Chung et al, 1993; Li et al, 1994; Zheng et al, 1994), so analysis of the p53 mutation pattern can be used as a clonal marker for the differential diagnosis of multiple cancers (Oda et al, 1992).

We identified three different distribution patterns of p53 mutations in the double lung tumours in this study: [A] only one tumour had the mutation, [B] each tumour had different mutation, and [C] the same mutation was found in both tumours. The cases of the [A] or [B] pattern were considered to be multicentric or double primary lung cancers, because patterns [A] and [B] indicated the heterogeneity of p53 mutation in the double tumours and suggested that each tumour arose from a different cellular clone. The cases of pattern [C] may be classified as the primary and intrapulmonary metastatic tumours. Another possibility is that cases of multicentric lung cancers could show pattern [C] by possessing the same mutation by chance. It is well known that there are mutational hotspots in the p53 gene (Hollstein et al, 1991; Taylor et al, 1994), the probability that the same mutation at codon 268 occurred independently in different tumours is, however, extremely low when the great variety of p53 mutations is considered.

In cases 19 and 20, the lymph node metastases were suggested to be derived from the smaller tumour because of the same mutational pattern of the p53 gene. p53 mutation may be a more important factor than the size of the tumour in metastasizing.

The mutational patterns and nucleotide substitutions in the p53 gene are specific to each organ. For instance, G:C $\rightarrow$A:T conversion is found in 63% of the p53 mutations in colon cancers, while its frequency is only 24% in lung cancers. In contrast, G:C$\rightarrow$T:A conversion is found only in 9% of the p53 mutations in colon cancers and 40% in lung cancers (Greenblatt et al, 1994). This suggests that different carcinogens and different mechanisms of p53 alterations occur in each organ. In our study, C$\rightarrow$T alterations accounted for six of eight (75%) independent mutations found in the multicentric cancers. There might be some particular events in the carcinogenesis of double primary lung cancer.

It is to be desired that the diagnosis of double primary lung cancer or pulmonary metastasis should be made before surgery if possible, because the clinical course and post-operative therapy may be changed by the diagnosis. Genes can be amplified by PCR from a very small amount of tissue obtained, for example, by a bronchial biopsy specimen at the time of bronchoscopy. Use of p53 alterations as a genetic marker to classify the double lung tumours may not be complete, because p53 mutations would not be found in all cases. However, there is a possibility that multicentric lung cancers and pulmonary metastasis can be differentiated by using p53 alterations, at least for a subset of lung tumours

© Cancer Research Campaign 1999 

with p53 gene mutations. Although the number of cases analysed was small, this study suggested that there were more multicentric cancer cases than the pulmonary metastatic cases in the double lung tumour cases. Our results support that the incidence of synchronous double primary lung cancer may be higher than predicted.

### REFERENCES

Caamano J, Ruggeri B, Momiki S, Sickler A, Zhang SY and Klein-Szanto AJP (1991) Detection of p53 in primary lung tumors and non-small cell lung carcinoma cell lines. *Am J Pathol* **139**: 839–845

Chiba I, Takahashi T, Nau MM, D'Amico D, Curiel DT, Mitsudomi T, Buchhagen DL, Carbone D, Piantadosi S and Koga H (1990) Mutations in the p53 gene are frequent in primary, resected non-small cell lung cancer. *Oncogene* **5**: 1603–1610

Chung KY, Mukhopadhyay T, Kim J, Casson A, Ro JY, Goepfert H, Hong WK and Roth JA (1993) Discordant p53 gene mutations in primary head and neck cancers and corresponding second primary cancers of the upper aerodigestive tract. *Cancer Res* **53**: 1676–1683

Ferguson MK, DeMeester TR, DesLauriers J, Little AG, Piraux M and Golomb H (1985) Diagnosis and management of synchronous lung cancers. *J Thorac Cardiovasc Surg* **89**: 378–385

Gasparotto D, Maestro R, Barzan L, Vukosavljevic T, Doglioni C, Sulfaro S, Piccinin S and Boiocchi M (1995) Recurrences and second primary tumors in the head and neck region: differentiation by p53 mutation analysis. *Ann Oncol* **6**: 933–939

Greenblatt MS, Bennett WP, Hollstein M and Harris CC (1994) Mutations in the p53 tumor suppressor gene: clues to cancer etiology and molecular pathogenesis. *Cancer Res* **54**: 4855–4878

Habuchi T, Takahashi R, Yamada H, Kakei Y, Sugiyama T and Yoshida O (1993) Metachronous multifocal development of urothelial cancers by intraluminal seeding. *Lancet* **342**: 1087–1088

Hollstein M, Sidransky D, Vogelstein B and Harris CC (1991) p53 mutations in human cancers. *Science* **253**: 49–53

Kandioler D, Dekan G, End A, Pasching E, Buchmayer H, Gnant M, Langmann F, Mannhalter C, Eckersberger F and Wolner E (1996) Molecular genetic differentiation between primary lung cancers and lung metastases of other tumors. *J Thorac Cardiovasc Surg* **111**: 827–832

Kishimoto Y, Murakami Y, Shiraishi M, Hayashi K and Sekiya T (1992) Aberrations of the p53 tumor suppressor gene in human non-small cell lung carcinomas of the lung. *Cancer Res* **52**: 4799–4804

Kunitoh H, Eguchi K, Yamada K, Tsuchiya R, Kaneko M, Moriyama N and Noguchi M (1992) Intrapulmonary sublesions detected before surgery in patients with lung cancer. *Cancer* **70**: 1876–1879

Li ZH, Zheng J, Weiss LM and Shibata D (1994) c-K-ras and p53 mutations occur very early in adenocarcinoma of the lung. *Am J Pathol* **144**: 303–309

Martini N and Melamed MR (1975) Multiple primary lung cancers. *J Thorac Cardiovasc Surg* **70**: 606–612

Mathisen DJ, Jensik RJ, Faber LP and Kittle FK (1984) Survival following resection for second and third primary lung cancers. *J Thorac Cardiovasc Surg* **88**: 502–510

Mitsudomi T, Steinberg SM, Nau MM, Carbone DD, Amico D, Bodner S, Oie HK, Linnoila RI, Mulshine JL and Minna JD (1992) p53 gene mutations in non small cell lung cancer cell lines and their correlation with the presence of ras mutations and clinical features. *Oncogene* **7**: 171–180

Mitsudomi T, Yatabe Y, Koshikawa T, Hatooka S, Shinoda M, Suyama M, Sugiura T, Ogawa M and Takahashi T (1997) Mutations of the p53 tumor suppressor gene as clonal marker for multiple primary lung cancers. *J Thorac Cardiovasc Surg* **114**: 354–360

Mok CH, Tsao SW, Knapp RC, Fishbaugh PM and Lau CC (1992) Unifocal origin of advanced human epithelial ovarian cancers. *Cancer Res* **52**: 5119–5122

Nakajima J, Furuse A, Oka T, Kohno T and Ohtsuka T (1996) Excellent survival in a subgroup of patients with intrapulmonary metastasis of lung cancer. *Ann Thorac Surg* **61**: 158–163

Naruke T, Goya T, Tsuchiya R and Suemasu K (1988) Prognosis and survival in resected lung carcinoma based on the new international staging system. *J Thorac Cardiovasc Surg* **96**: 440–447

Nees M, Homann N, Discher H, Andl T, Enders C, Herold MC, Schuhmann A and Bosch FX (1993) Expression of mutated p53 occurs in tumor-distant epithelia of head and neck cancer patients: a possible molecular basis for the development of multiple tumors. *Cancer Res* **53**: 4189–4196

Oda T, Tsuda H, Scarpa A, Sakamoto M and Hirohashi S (1992) Mutation pattern of the p53 gene as a diagnostic marker for multiple hepatocellular carcinoma. *Cancer Res* **52**: 3674–3678

Reichel MB, Ohgaki H, Petersen I and Kleihues P (1994) p53 mutations in primary human lung tumors and their metastases. *Mol Carcinog* **9**: 105–109

Schlegel U, Rosenfeld MR, Volkenandt M, Rosenblum M, Dalmau J and Furneaux H (1992) p53 gene mutations in primary lung tumors are conserved in brain metastases. *J Neurooncol* **14**: 93–100

Sozzi G, Miozzo M, Pastorino U, Pilotti S, Donghi R, Giarola M, Gregorio LD, Manenti G, Radice P, Minoletti F, Porta GD and Pierotti MA (1995) Genetic evidence for an independent origin of multiple preneoplastic and neoplastic lung lesions. *Cancer Res* **55**: 135–140

Taylor JA, Watson MA, Devereux TR, Michels RY, Saccomanno G and Anderson M (1994) p53 mutation hotspot in radon-associated lung cancer. *Lancet* **343**: 86–87

Yokota J and Sugimura T (1993) Multiple steps in carcinogenesis involving alterations of multiple tumor suppressor genes. *FASEB J* **7**: 920–925

Zheng J, Shu Q, Li ZH, Tsao JI, Weiss LM and Shibata D (1994) Patterns of p53 mutations in squamous cell carcinoma of the lung. *Am J Pathol* **145**: 1444–1449

*© Cancer Research Campaign 1999*
